# Reply to Lei and Koulaouzidis

**DOI:** 10.1055/a-2866-5797

**Published:** 2026-07-30

**Authors:** James Turvill, Monica Haritakis, Scott Pygall, Emily Bryant, Harriet Cox, Greg Forshaw, Crispin Musicha, Victoria Allgar, Robert Logan, Mark McAlindon

**Affiliations:** 1 Department of Gastroenterology 8749 York and Scarborough Teaching Hospitals NHS Foundation TrustYorkEnglandUnited Kingdom of Great Britain and Northern Ireland; 2 Research and Innovation 8749 York and Scarborough Teaching Hospitals NHS Foundation TrustYorkEnglandUnited Kingdom of Great Britain and Northern Ireland; 3 NHS Cancer Programme 2394 NHS EnglandRedditchEnglandUnited Kingdom of Great Britain and Northern Ireland; 4 Faculty of Health, Medical Statistics Group 6633 Plymouth UniversityPlymouthEnglandUnited Kingdom of Great Britain and Northern Ireland; 5 National Specialty Adviser for Endoscopy and Screening 2394 NHS EnglandRedditchEnglandUnited Kingdom of Great Britain and Northern Ireland; 6 Academic Unit of Gastroenterology and Hepatology 7318 Sheffield Teaching Hospitals NHS Foundation TrustSheffieldUnited Kingdom of Great Britain and Northern Ireland



Lei and Koulaouzidis have largely and succinctly enunciated the colon capsule endoscopy (CCE) conundrum and way forward in their letter. Thank you. One element of that is to ensure the systematic follow-up linkage of discharged or deferred patients. Clearly this is essential and such an approach has driven up standards in colonoscopy, many lessons from which will be transferrable into an optimized CCE for the future. Sadly, data collection for longer term follow-up was not funded within this evaluation, but efforts are underway, through establishment of a registry, to collate the English experience.

Follow-up data will also inform other observations made that might influence the longer-term positioning of CCE within surveillance. First, because colonoscopy is the reference standard, polyps identified at CCE and then missed at colonoscopy are labelled false positives. Double counting is clearly a phenomenon, but will diminish with experience and technology. However, we find it difficult to conclude that all of those larger polyps identified were falsely so. Time will tell. Second, the criteria for endoscopic follow-up were cautious and empiric. If guidance were to change, or perhaps be more patient-centered, the picture would look a little different.

This brings us to the final point, which is that of choice. We would argue that choice is closely linked to engagement. These are important factors for us to consider because they are for people with bowel disease.

**Publication note**

Letters to the editor do not necessarily represent the opinion of the editor or publisher. The editor and publisher reserve the right to not publish letters to the editor, or to publish them abbreviated or in extracts.

